# Delays in tuberculosis diagnosis and treatment in India: A patient journey analysis from Mumbai and Patna during the COVID-19 Pandemic

**DOI:** 10.1371/journal.pgph.0007214

**Published:** 2026-09-16

**Authors:** Mohammad Abdullah Heel Kafi, Poshan Thapa, Madhukar Pai, Charity Oga-Omenka

**Affiliations:** 1 Cardiac Services BC, Provincial Health Service Authority, British Columbia, Canada; 2 Research Institute of the McGill University Health Centre, Montréal, Quebec, Canada; 3 McGill International TB Centre, Montreal, Canada; 4 Department of Epidemiology, Biostatistics and Occupational Health, McGill University, Montreal, Canada; 5 School of Public Health Sciences, University of Waterloo, Waterloo, Canada; Dr D Y Patil Medical College Hospital and Research Centre, INDIA

## Abstract

Tuberculosis (TB) care for people with TB in India are often fragmented, undermining timely diagnosis and treatment. Understanding patient journeys is critical to strengthening TB care delivery and advancing elimination goals. We conducted a cross-sectional study of 400 people with TB diagnosed between 2020–2022 in two Indian cities: Mumbai (n = 200) and Patna (n = 200). Using structured interviews, we assessed care-seeking behavior, delays to diagnosis and treatment, the number and type of healthcare encounters, and out-of-pocket costs. Participants predominantly initiated care in the private sector (Mumbai 91%; Patna 85%), often at pharmacies or private clinics. Care journeys were fragmented, with multiple provider visits before diagnosis. The median total delay from symptom onset to treatment initiation was 35 days (IQR 13–81) in Patna and 26 days (IQR 12–59) in Mumbai. Provider delays contributed a median of ~19 days in both cities. Participants made a median of 3 pre-diagnosis healthcare visits, and 23% had ≥ 6 encounters. The financial burden was substantial particularly in Mumbai, where consultation and diagnostic costs were higher than in Patna. Longer delays and more encounters were associated with male sex, unemployment, larger household size, and care-seeking hesitancy during the study period. Patient journeys in urban India during the COVID-19 pandemic were prolonged and costly, with delays driven primarily by systemic fragmentation rather than patient hesitancy. The reliance on an under-regulated private sector led to multiple pre-diagnosis encounters (23% requiring more than 6) and significant out-of-pocket costs. Because no individual characteristic consistently predicted delay, these findings suggest that structural reforms including standardized diagnostic pathways and enhanced public-private integration are essential to build health system resilience and accelerate TB elimination.

## Introduction

Tuberculosis (TB), an infectious disease caused by Mycobacterium tuberculosis, remains a significant public health issue, with India accounting for 26% of the global TB burden [1]. In 2022, approximately 2.77 million TB cases were reported in the country [1,2]. However, undiagnosed or “missing” TB cases remain a significant challenge, with up to 25% of cases unreported, further adding to transmission and disease burden [3]. The slow decline in TB incidence rates is primarily attributed to persistent socio-economic factors such as poverty, malnutrition, overcrowded living conditions, and limited access to healthcare services. Compounding these challenges is a large, under-regulated, and diverse private sector, which ranges from small clinics to multi-specialty hospitals and includes informal healthcare providers and highly qualified specialists, catering to up to 50% of people with TB in India [4–7]. Furthermore, health system disruptions can reveal critical vulnerabilities in TB care delivery, particularly in densely populated countries like India.

This study examines the COVID-19 pandemic serves as a case study of health system disruptions, with lessons that remain relevant for strengthening TB services against future crises. The pandemic exacerbated pre-existing challenges, severely disrupting TB treatment services, case detection, and reporting processes [8,9]. National lockdowns, social distancing measures, and the diversion of healthcare resources towards COVID-19 management significantly reduced the availability of diagnostic and treatment services for TB [10–12]. For instance, TB case notifications in India dropped by 25% in 2020 compared to the previous year, with the most substantial decline observed during the nationwide lockdown from March to April [13].This decline in case detection corresponded with an 11% increase in TB-related mortality in 2020 compared to 2019 [14]. These disruptions were even more pronounced in India’s private healthcare sector, which accounts for an estimated 60–85% of initial TB care interactions occur, resulting in a 44% decline in case notifications during the lockdown period [11].

Understanding the systemic weaknesses in TB care revealed during the pandemic requires a comprehensive examination of patients’ healthcare-seeking behaviors and their journeys to diagnosis and treatment. In this context, Patient Journey Analyses (PJAs) offer a valuable approach for identifying delays in diagnosis, treatment, and healthcare visits, as well as exploring systemic barriers that hinder access to timely care [15–18]. While the Care Cascade measures population-level attrition [19] and Patient Pathway Analysis (PPA) [20] primarily evaluates the geographic alignment of services, PJA focuses on the individual-level chronology of the care-seeking experience [21,22]. By mapping every healthcare encounter from symptom onset, PJA allows for the measurement of specific delay durations and the identification of ‘missed opportunities’ for diagnosis that are frequently obscured in aggregate data. This analytical approach is particularly valuable for identifying persistent vulnerabilities even as health systems recover from major disruptions. While conventional PPAs identify where cases are missed, they often lack the capacity to measure the specific duration of delays or capture the individual-level factors, such as financial standing or employment status, associated with prolonged pathways. By utilizing PJA, this study moves beyond aggregate notification trends to examine how the COVID-19 pandemic added new layers of complexity to these individual trajectories, providing a more granular understanding of the barriers to TB care in urban India.

Despite the well-documented impact of COVID-19 on TB case detection, there remains a paucity of research exploring the specific delays and barriers encountered by people with TB during the pandemic, particularly in densely populated urban centers in India. While previous studies have primarily focused on aggregate changes in TB notification rates [23,24] or provided granular insights into patient care journeys during non-pandemic periods [25–28], there remains a lack of evidence regarding how health system shocks during the COVID-19 pandemic specifically altered these journeys. By focusing on individual experiences in two contrasting urban centers, this study addresses a critical gap in understanding how pandemic-related barriers translated into fragmented healthcare interactions and prolonged delays.

Mumbai and Patna were purposefully selected for this study to capture contrasting healthcare situations in India during the COVID-19 pandemic. Mumbai, one of the most densely populated cities globally, reported over 85,000 COVID-19 cases by mid-2020 and became an early epicenter of the pandemic in India, resulting in significant strain on its healthcare infrastructure [29]. Mumbai faces an exceptional TB burden, particularly in its high-density informal settlements. The Dharavi slum, for instance, houses approximately 1 million residents within only 0.8 square miles and reports roughly 300 new drug-resistant TB (DRTB) cases annually, representing one of the highest concentrations of DRTB globally. This setting was already characterized by significant baseline vulnerabilities, including a pre-pandemic loss-to-follow-up rate of 18% and treatment completion rates for DRTB often falling below 50% [30]. These challenges were compounded during the pandemic, which saw a 28% to 69% decline in notifications and significant population displacement, with an estimated 26% of people on DRTB treatment relocating [30,31]. In contrast, Patna, the capital of Bihar, represents a setting with a weaker baseline healthcare infrastructure and historically lower density of healthcare providers. This vulnerability was acutely exposed during the pandemic; while the national average for TB notification declines was 25%, Bihar recorded an 80% reduction during lockdown phases. Furthermore, Patna faced a unique system-level shock due to massive reverse migration, as thousands of workers returned from urban centers to rural Bihar, placing an unprecedented strain on limited diagnostic services [32].

By examining patient journeys in these two settings, this study aims to uncover delays in diagnosis and treatment, the frequency of healthcare interactions required for diagnosis, and the factors associated with delays in care during the COVID-19 pandemic. These findings provide evidence to inform approaches to building more resilient health system, highlighting where targeted interventions can strengthen TB care against existing challenges and future disruptions.

## Methods

### Study design and setting

This cross-sectional study was conducted in Patna (February–March 2022) and Mumbai (December 2022–January 2023). Although data collection occurred at different points in time, the study period for participant eligibility was standardized to 2020–2022 in both sites, ensuring that both cohorts provided reflections on journeys occurring during the same window of peak pandemic disruption and subsequent health system recovery. While formal COVID-19 restrictions in Mumbai were lifted in April 2022, capturing this recovery period was essential to demonstrate how diagnostic and treatment delays reflect structural weaknesses that persist beyond the acute crisis period. This study was part of a larger mixed-methods study conducted in Nigeria, Indonesia, and India, aimed at identifying disruptions to TB care during the COVID-19 pandemic and identifying health system adaptations that could strengthen resilience. Findings from this broader study have already been reported in several publications [22,33–35].This manuscript focuses on the quantitative aspects of a broader study, specifically examining tuberculosis (TB) diagnosis and treatment delays, as well as the factors contributing to these delays during the COVID-19 pandemic. Qualitative findings from a subset of participants will be reported in a forthcoming manuscript. The study in India was facilitated by the ISERDD, a research organization with an established presence in urban health across multiple regions of India, including Mumbai and Patna.

### Study participants and sampling

As part of a broader multi-country research effort examining TB care disruptions in India, Nigeria, and Indonesia, a target sample size of 400 participants (200 per city) was selected for the Indian component. This sample size was determined to be logistically feasible for intensive, in-person patient journey mapping while providing sufficient descriptive data to compare healthcare-seeking patterns across the two contrasting urban settings of Mumbai and Patna. Participants were eligible if they were 18 years old or older and had sought healthcare services for symptoms suggestive of TB or had been diagnosed with TB during the COVID-19 pandemic period. Study recruitment focused on patients diagnosed with TB during the COVID-19 pandemic (2020–2022). A purposive sampling strategy was employed to ensure participants represented diverse healthcare interactions, including those who accessed care from both the public and private sectors. Recruitment was conducted by trained field officers affiliated with ISERDD, who also had prior experience with community-based TB research. Research supervisors identified eligible participants through clinic registries and direct approach during follow-up visits at a mix of private outpatient clinics and public hospitals in both cities. This approach ensured that the sample captured diverse care-seeking trajectories, including those who transitioned between sectors (e.g., from private diagnosis to public treatment initiation). Regarding clinical characteristics, the sample comprised predominantly drug-susceptible TB (DS-TB) cases; while drug-resistant TB (DR-TB) cases were not excluded, their smaller proportion reflects the overall epidemiological distribution at study sites. This analysis focused primarily on pre-treatment diagnostic delays, where barriers are broadly similar across TB types. We acknowledge that DR-TB treatment involves longer durations and higher costs; however, this analysis focused primarily on pre-treatment diagnostic delays, where barriers are broadly similar across TB types.

### Outcomes of interest and definitions

**Fig 1:**
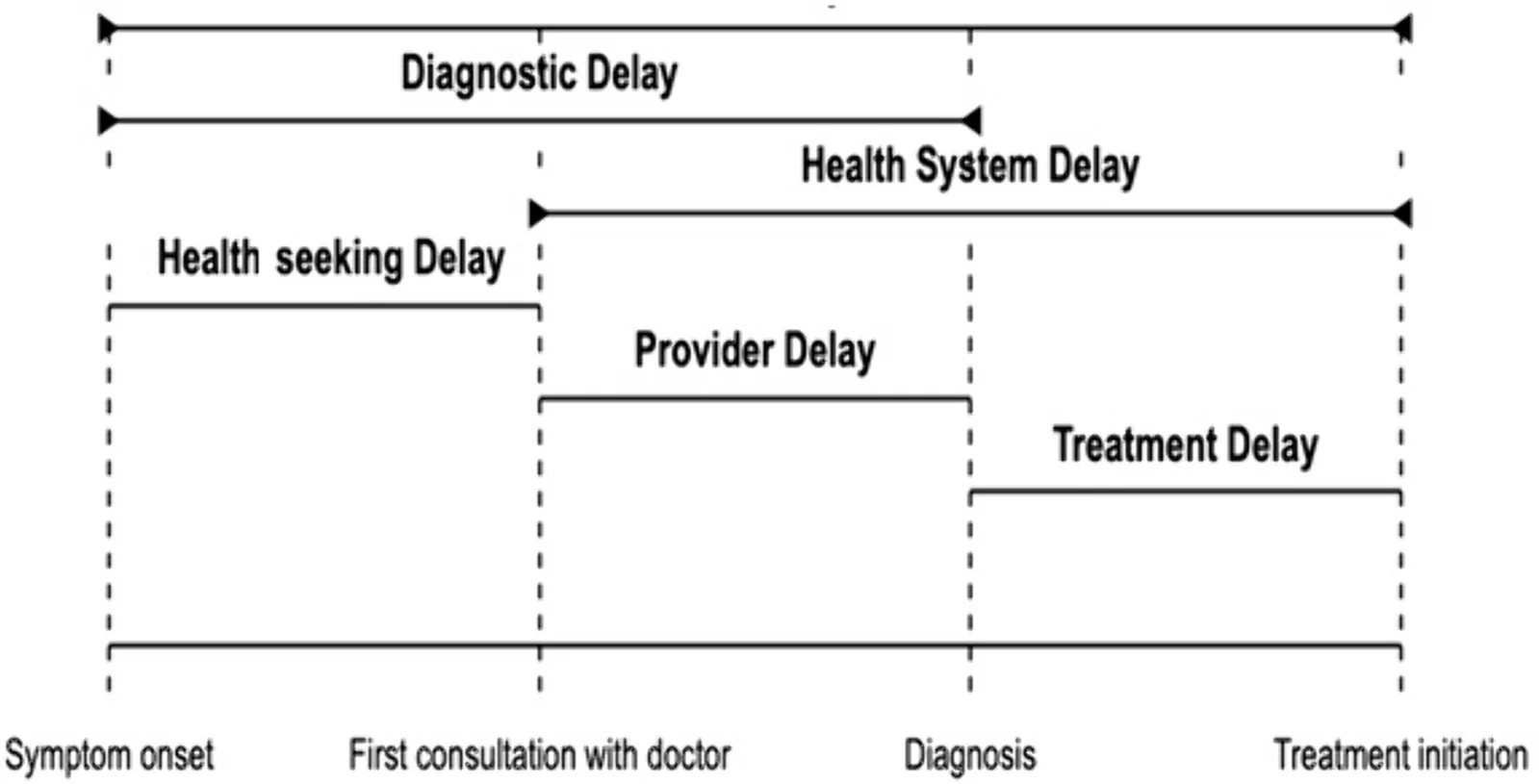
Definition of delays in healthcare-seeking, diagnosis, and treatment, figure recreated for clarity, adapted from the World Health Organisation.

The primary outcomes were: 1) delays in TB diagnosis and treatment initiation, and 2) the type and number of healthcare encounters involved in the patient’s care-seeking journey. Fig 1 outlines the different types of delays: (i) Health-seeking delay is defined as the number of days from the onset of symptoms to the first healthcare consultation; (ii) Provider delay is defined as the number of days from the first consultation to TB diagnosis. We acknowledge that in the Indian context, this phase often includes ‘patient-side’ delays, where individuals may be unable to return for follow-up testing immediately due to job insecurity, financial constraints, or a lack of familial support for travel. and (iii) Treatment delay is defined as the number of days from diagnosis to treatment initiation [36,37]. An encounter was defined as any interaction with a healthcare provider, including visits to pharmacists, clinics, hospitals, informal providers, and specialists. The study also assessed the frequency and patterns of healthcare visits as well as the types of healthcare facilities assessed, including private clinics, public hospitals, and pharmacies.

This study collected all delay measurements through structured patient interviews using a standardized questionnaire administered by trained research assistants. People with TB were asked to recall the dates when their symptoms began, their first healthcare contact, and when TB diagnosis and treatment were initiated. Interviewers used calendar prompts to improve recall accuracy for participants who were unable to recall exact dates. For encounter information from the patient, all healthcare visits between symptom onset and treatment initiation were documented, including the type of provider, diagnostic tests performed, and recommendations received.

### Study tool and data collection

The data collection tool was developed based on established Patient Pathway Analysis (PPA) and Patient Journey Analysis (PJA) frameworks. To ensure the tool was appropriately adapted to the Indian urban context, we consulted several seminal Indian care-pathway studies—including Atre et al. (2022), Lobo et al. (2018), Bhattacharya et al. (2019), and Mistry et al. (2016)—during the design phase [25–27,38]. The tool was further adapted to capture specific COVID-19-related healthcare disruptions and behavioral shifts. The structured questionnaire contained sections on socio-demographic characteristics, symptom history, healthcare-seeking behavior, diagnostic experiences, treatment initiation, and COVID-19-related barriers and disruptions. The tool was translated into the local language (Hindi) and then back translated to ensure linguistic accuracy. It was pre-tested with a few people with TB not included in the final sample, which led to refinements in question phrasing and response categories. The final version of the questionnaire is provided in the supporting information (Table A in S1 Appendix).

A team of trained research assistants with public health backgrounds and prior TB research experience conducted data collection. All data collectors received training on study procedures, questionnaire administration, and research ethics. Interviews were conducted in-person at TB treatment centers in private settings to ensure participant confidentiality and encourage open disclosure of sensitive health and financial information. All data were collected using encrypted, tablet-based electronic forms (ODK or similar platform) to minimize data handling errors and maintain data integrity. Furthermore, all research assistants underwent specific training in ethical interviewing techniques, emphasizing the right of participants to withdraw at any time or decline to answer specific questions without affecting their care. The study coordinator performed weekly quality checks to identify and address data inconsistencies or missing information. To minimize recall bias, interviewers used calendar tools and referred to significant personal and national events to help participants accurately recall the dates and details of their healthcare interactions.

### Statistical analysis

All data cleaning, visualization, and statistical analysis were performed using statistical software R version 4.4.1 [39]. Numerical variables were described by median and interquartile range (IQR), and categorical variables were described using proportion (percentage). Due to the skewed distribution of continuous outcomes and the presence of outliers, quantile regression (tau = 0.5) was employed to investigate the relationship between the outcome variables (various delays) and potential predictors. Linear regression was used to analyze the number of healthcare encounters.

Univariate regression was initially used to explore factors associated with delays and the number of encounters. Multivariate models were then fitted to control for confounders and to examine the impact of COVID-19 on the outcomes of interest. Variables considered in the multivariate analysis were selected based on previous literature [40] and systematic reviews summarising risk factors for delays in TB care [41–44], which included age, gender, marital status, household size, education level, employment status, and financial standing, as well as clinical and exposure-related factors such as contact history with active TB cases, having friends or family with COVID-19, and whether COVID-19 affected the willingness to seek healthcare. Additional factors, such as distance to the nearest public and private healthcare facilities to explore geographic accessibility in delays, as well as internet usage history, which may reflect differential access to healthcare information during the pandemic, were included in the outcome related to the number of encounters [41–44].

Sensitivity analyses were conducted using logistic regression models to validate findings from the quantile regression and assess the robustness of associations. A cut-off of 30 days was used to define a health-seeking delay, and a cut-off of six encounters was used to indicate a high number of healthcare interactions, based on the same set of predictors as described above. The 30-day threshold has been widely adopted across Indian and global TB pathway studies as the standard cut-off for prolonged health-seeking delays, and delays exceeding one month have been consistently associated with increased transmission risk and worse clinical outcomes [42,45,46]. The six-encounter cut-off was selected based on the distribution in our data and reflects a point beyond which fragmented care-seeking is associated with substantial financial burden; Indian studies consistently show that multiple pre-diagnosis provider visits are a primary driver of catastrophic out-of-pocket costs, even where public sector treatment is free [47,48]. These thresholds were used in subsequent analyses of factors associated with delays in the TB care journey.

### Ethics statement

This study was funded by the Bill and Melinda Gates Foundation (INV022420). Ethical approval was obtained from the McGill University Research Ethics Board (Covid BMGF / 2021–7197), the Georgetown-Medstar IRB (STUDY00003422), and the Institute for Social and Economic Research on Development and Democracy (ISERDD). The recruitment process ensured that participants provided informed consent before data collection. Specifically, written informed consent was obtained from all participants prior to the interview. For participants who were unable to read, the consent form was read aloud by a trained field officer and verbal consent was documented by a witness signature on the consent form. No minors were enrolled; all participants were 18 years of age or older.

## Results

### Participants baseline characteristics

The baseline characteristics of the study participants from Patna and Mumbai, presented in Table 1, showed distinct demographic and healthcare access patterns in both cities. In Patna, the mean age of participants was 39.1 years, with 60% being male; in Mumbai, the mean age was 31.6 years, with 63% being female. Marital status also varied, with a higher proportion of single individuals in Mumbai (43.5%) compared to Patna (30%). Participants in Patna reported slightly larger household sizes (mean = 4.68, SD = 2.44) than those in Mumbai (mean = 4.09, SD = 2.31). Educational attainment was notably higher among Mumbai participants, with 23% having completed a bachelor’s degree, compared to 14.5% in Patna. Employment status was similar across both cities, with more than half of participants being unemployed or underemployed (52% in Patna and 54.5% in Mumbai). Financial adequacy was reported by approximately half of the participants in both Patna (46.5%) and Mumbai (53.5%). It should be noted that the employment data reflect participants’ status at the time of the interview and do not distinguish between pre-existing unemployment and job loss attributable to TB illness or pandemic-related disruptions.

**Table 1:** Baseline characteristics of the study sample (N = 400).

Characteristics	Patna (N = 200)	Mumbai (N = 200)
**Age**		
Mean (SD)	39.1(15.9)	31.6 (11.0)
Missing		6 (3.0%)
**Gender**		
Male	120 (60%)	70 (35.0%)
Female	80 (40%)	126 (63.0%)
Missing		4 (2.0%)
**Marital status**		
Single	60 (30%)	87 (43.5%)
Married	118 (59%)	104 (52.0%)
Widowed	22 (11%)	0 (0%)
Missing		4 (2.0%)
**Individuals in house**		
Mean (SD)	4.68 (2.44)	4.09 (2.31)
Missing		4 (2.0%)
**Highest education level**		
Primary 6 or lower	60 (30%)	31 (15.5%)
Junior secondary school	49 (24.5%)	50 (25.0%)
Senior secondary school	18 (9%)	51 (25.5%)
Bachelor’s degree	29 (14.5%)	46 (23.0%)
Post-graduate certificate	9 (4.5%)	10 (5.0%)
Other	33 (16.5%)	7 (3.5%)
Missing		4 (2.0%)
**Employment**		
Working	60 (30%)	80 (40.0%)
Self-employed	30 (15%)	6 (3.0%)
Unemployed/underemployed	104 (52%)	109 (54.5%)
Missing	6 (3%)	4 (2.0%)
**Financial standing**		
Adequate	93 (46.5%)	107 (53.5%)
Barely adequate	72 (36%)	73 (36.5%)
Inadequate	35 (17.5%)	15 (7.5%)
Missing		5 (2.5%)

### Healthcare access and TB status

Most participants in both cities had good access to healthcare facilities, with 63.5% of participants in Patna and 79.5% in Mumbai living within 2 km of a public healthcare center. Similarly, most participants in both cities reported proximity to private health centers, with 96.5% of participants in Patna and 94.5% in Mumbai residing within 2 km of a private facility (Table 2).

**Table 2:** Distribution of study participants based on TB-related characteristics and healthcare Access.

Characteristics	Patna (N = 200)	Mumbai (N = 200)
**Nearest public health center**		
Less than 2 km away	127 (63.5%)	159 (79.5%)
Between 2–10 km away	71 (35.5%)	32 (16.0%)
Over 10km	2 (1%)	2 (1.0%)
Do not know/not sure		2 (1.0%)
Missing		5 (2.5%)
**Nearest private health center**		
Less than 2 km away	193 (96.5%)	189 (94.5%)
Between 2–10 km away	7 (3.5%)	5 (2.5%)
Over 10km		0 (0%)
Missing		6 (3.0%)
**Symptoms that prompted a visit to a healthcare provider** ^**a**^		
Cough	175 (87.5%)	121 (60.5%)
Chest congestion	93 (46.5%)	39 (19.5%)
Head congestion	50 (25.0%)	20 (10.0%)
Sore throat	72 (36.0%)	18 (9.0%)
Rashes or allergies	14 (7.0%)	3 (1.5%)
Trouble breathing/wheezing	90 (45.0%)	44 (22.0%)
Weight loss	154 (77.0%)	92 (46.0%)
Night sweats	59 (29.5%)	15 (7.5%)
Fever	155 (77.5%)	124 (62.0%)
Lack of appetite	143 (71.5%)	85 (42.5%)
**TB care stage now**		
Currently taking TB medication	76 (38.0%)	110 (55.0%)
Completed all TB medication	108 (54.0%)	85 (42.5%)
**Location of TB diagnosis**		
Public Hospital	22 (11.0%)	2 (1.0%)
Private outpatient clinic, health center, or dispensary	172 (86.0%)	86 (43.0%)
Private hospital or nursing home	5 (2.5%)	107 (53.5%)
**Diagnosis is given in the same location as the first encounter**		
No	105 (52.5%)	144 (72.0%)
Yes	95 (47.5%)	52 (26.0%)
Missing		4 (2.0%)
**Location of TB Treatment**		
Community health worker/ Community Health Extension Worker (CHEW)	1 (0.5%)	2 (1.0%)
Public Hospital	24 (12.0%)	5 (2.5%)
Private outpatient clinic, health center, or dispensary	170 (85.0%)	60 (30.0%)
Private hospital or nursing home	5 (2.5%)	129 (64.5%)
**Treatment is given in the same location as the site of diagnosis**		
No	53 (26.5%)	76 (38.0%)
Yes	147 (73.5%)	120 (60.0%)
Missing		4 (2.0%)

^a^Participants could select multiple symptoms; percentages do not sum to 100%.

Symptoms prompting healthcare visits differed between the two cities, with cough, weight loss, and fever being more frequently reported in Patna (87.5%, 77%, and 77.5%, respectively) compared to Mumbai (60.5%, 46%, and 62%). A higher proportion of participants in Mumbai were still taking TB medication at the time of the survey (55%) compared to Patna (38%). In Patna, 86% of participants were diagnosed at private outpatient clinics, while 53.5% of participants in Mumbai were diagnosed at private hospitals or nursing homes. Similarly, treatment was predominantly administered at private outpatient clinics in Patna (85%), whereas 64.5% of participants in Mumbai received treatment at private hospitals or nursing homes. More details of the symptoms that led patients to seek care from various providers are presented in the supporting information (Table B in S1 Appendix).

### Distribution of delays during COVID-19

**Fig 2:**
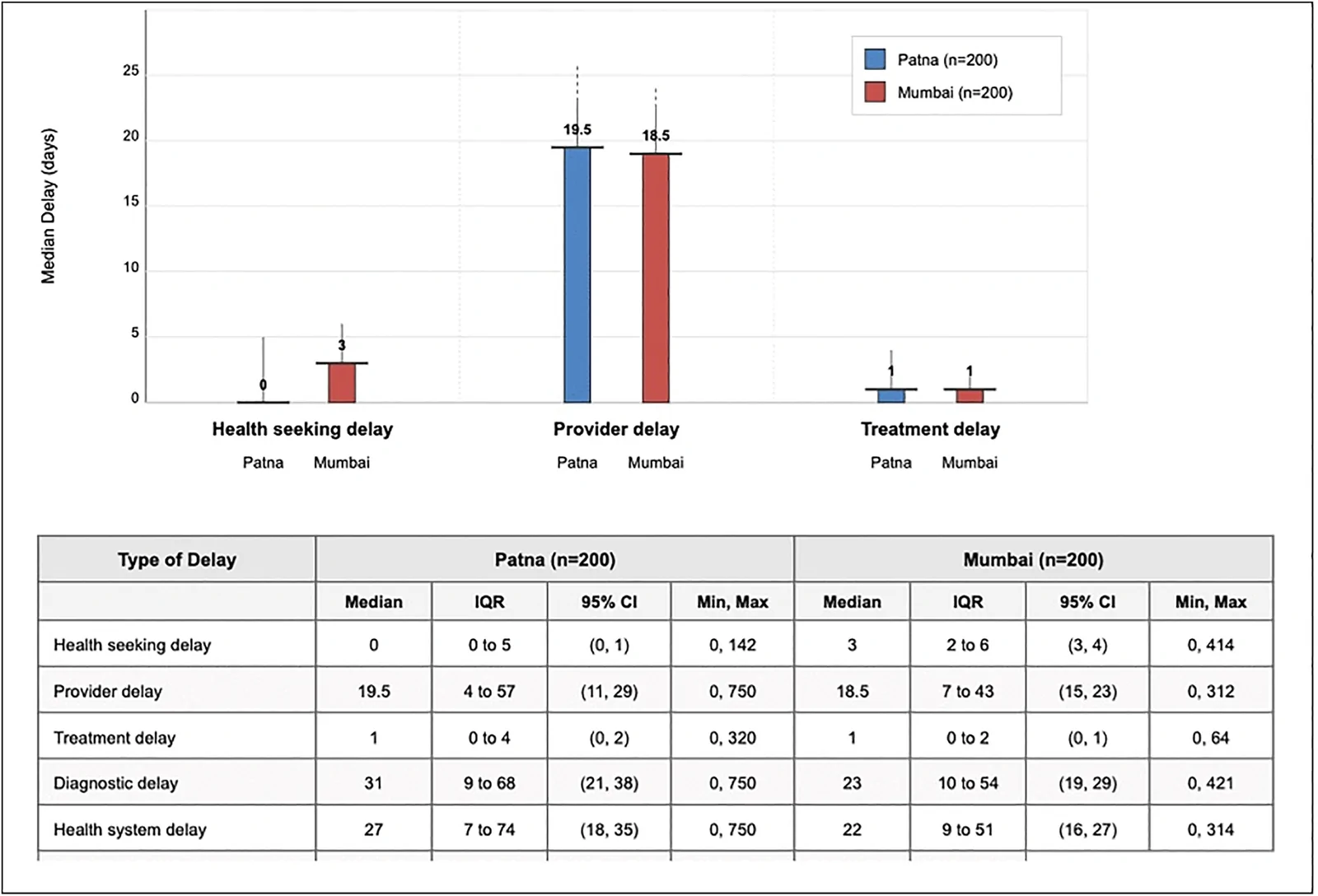
TB Diagnostic and Health System Delays During COVID-19 in Patna and Mumbai.

The distribution of delays experienced by people with TB during the COVID-19 pandemic in Patna and Mumbai is detailed in Fig 2**.** The median health-seeking delay was 0 days in Patna (IQR 0–5 days) compared to 3 days in Mumbai (IQR 2–6 days). These figures are notably shorter than the 1–2-month health-seeking delays reported in pre-pandemic Indian studies and likely reflect the overlap of TB symptoms (cough, fever) with COVID-19 symptoms during the study period, combined with intensified public health messaging encouraging immediate care-seeking for respiratory illness. Furthermore, 50.5% of Mumbai participants and 31.5% of Patna participants reported being more willing to seek care specifically because of the pandemic. The high density of community pharmacies, serving as a first point of contact for 41% of Patna participants, 96.5% of whom lived within 2 km of a private provider, further enabled near-immediate care-seeking without a formal appointment. Provider delays showed similarity in both cities, with 19.5 days in Patna (IQR 4–57 days) and 18.5 days in Mumbai (IQR 7–43 days). However, diagnostic delays were notably longer in Patna, with a median of 31 days (IQR, 9–68), compared to 23 days in Mumbai (IQR, 10–54 days). Treatment delays were minimal in both cities, with a median of 1 day suggesting that treatment initiation was relatively prompt following a diagnosis. Health system delays, which encompass cumulative delays within the healthcare system, had a median of 27 days in Patna (IQR, 7–74 days) and 22 days in Mumbai (IQR, 9–51 days). The total delay, from initial health-seeking to treatment initiation, was longer in Patna, with a median of 35 days (IQR, 13–81 days), compared to 26 days in Mumbai (IQR, 12–59 days).

### Impact of COVID-19 on TB care utilization and associated cost

The impact of the COVID-19 on TB healthcare and utilization exposed distinct challenges in Patna and Mumbai, as presented in Table 3. Healthcare access disparities were particularly pronounced, with 14.5% of participants in Patna unable to reach a doctor due to facility closures, compared to just 4% in Mumbai. Similarly, medication access was disrupted for 4% of participants in Patna and only 0.5% in Mumbai. Movement restrictions further hindered access, preventing 12% of participants in Patna and 8% of participants in Mumbai from seeking care. However, prolonged waiting times were more commonly reported in Mumbai (21.5%) than in Patna (9.5%).

**Table 3:** The Impact of the COVID-19 Pandemic on TB Healthcare Utilization and Cost.

Characteristic	Patna (N = 200)	Mumbai (N = 200)
**Perspectives on COVID-19 and Healthcare Access**		
**Healthcare access impacts**		
Unable to reach a doctor because facilities were closed	29 (14.5%)	8 (4.0%)
Unable to receive medication because drug stores were closed	8 (4.0%)	1 (0.5%)
Obliged to seek care from a different facility than preferred	7 (3.5%)	3 (1.5%)
Waiting time became longer than normal/expected	19 (9.5%)	43 (21.5%)
Unable to leave the house to seek care due to movement restrictions	24 (12.0%)	16 (8.0%)
Other effects on healthcare	4 (2.0%)	132 ^a^(66.0%)
**Friends/family members diagnosed with COVID**		
Yes	25 (12.5%)	33 (16.5%)
**Willingness to seek healthcare**		
More willing	63 (31.5%)	101 (50.5%)
Less willing	19 (9.5%)	10 (5.0%)
No effect	118 (59.0%)	83 (41.5%)
**Reasons for increased willingness**		
Early healthcare seeking	58 (29.0%)	100 (50.0%)
Awareness of family member’s infection	26 (13.0%)	6 (3.0%)
Awareness of getting infected by others	17 (8.5%)	6 (3.0%)
Understanding of underlying/chronic condition	17 (8.5%)	–
**Healthcare Utilization Patterns**		
Types of contacted health providers ^b^		
Community pharmacy, chemist, drug shop, patent medicine vendor	105 (52.5%)	3 (1.5%)
Private laboratory	34 (17.0%)	1 (0.5%)
Public outpatient clinic (e.g., PHC/Health Post/Dispensary)	5 (2.5%)	5 (2.5%)
Public Hospital	59 (29.5%)	44 (22.0%)
Private outpatient clinic, health center, or dispensary	181 (90.5%)	182 (91.0%)
Private hospital or nursing home	6 (3.0%)	120 (60.0%)
**First source for treatment seeking**		
Community pharmacy, chemist, drug shop, patent medicine vendor	82 (41.0%)	–
Public Hospital	12 (6.0%)	2 (1.0%)
Private outpatient clinic, health center, or dispensary	103 (51.5%)	184 (92.0%)
Private hospital or nursing home	2 (1.0%)	5 (2.5%)
**Unmet healthcare needs**		
No	181 (90.5%)	191 (95.5%)
Yes	19 (9.5%)	5 (2.5%)
**Reason for unmet healthcare needs**		
Healthcare was not available because the practice was closed due to COVID-19	13 (6.5%)	3 (1.5%)
**Telemedicine use before the pandemic**		
Did not use telemedicine	191 (95.5%)	194 (97.0%)
**Telemedicine use since pandemic start**		
Did not use telemedicine	181 (90.5%)	188 (94.0%)
Once a month or less	11 (5.5%)	5 (2.5%)
**Healthcare Costs**		
Current healthcare costs compared to before COVID-19		
Costs stayed the same	50 (25.0%)	19 (9.5%)
Costs increased	127 (63.5%)	171 (85.5%)
**Consultation fee at this facility (Indian rupees)**		
Average fee (SD)	326 (237)	564 (461)
**Lab test price at this facility (Indian rupees)**		
Average price (SD)	2550 (3110)	9970 (12600)
**Monthly TB medication price (Indian rupees)**		
Average price (SD)	188 (660)	114 (372)

^a^In Mumbai, *Other effects* (66%) largely denote the requirement for mandatory COVID-19 screening prior to TB consultation and the temporary repurposing of primary health posts for pandemic response.”

^b^Participants could go multiple providers; percentages do not sum to 100%.

Behavioral changes related to healthcare-seeking were more pronounced in Mumbai, where 50.5% of participants reported an increased willingness to seek healthcare, largely due to early recognition of symptoms (50%). In Patna, 31.5% of participants reported an increased willingness to seek care.

The adaptability of the healthcare system was evident, with private outpatient clinics serving as the primary source of TB care in both cities (90.5% in Patna and 91% in Mumbai). However, there was a stark contrast in the use of private hospitals, with 60% of participants in Mumbai utilizing private hospitals or nursing homes, compared to only 3% in Patna. Unmet healthcare needs were reported by 9.5% of participants in Patna, compared to 2.5% in Mumbai, with facility closure being the primary reason for unmet needs. Despite the increased need for alternative healthcare service options during the pandemic, telemedicine use remained minimal, with only slight increases observed in both cities (5.5% in Patna and 2.5% in Mumbai).

A significant proportion of participants in both cities experienced increased healthcare costs, with 63.5% of participants in Patna and 85.5% in Mumbai reported higher expenses compared to pre-pandemic levels. Healthcare expenses in Mumbai were notably higher across various categories than in Patna. The average consultation fee in Mumbai was 564 Indian Rupees (SD = 461), nearly double that of Patna, at 326 Rupees (SD = 237). Similarly, the cost of lab tests was significantly higher in Mumbai, averaging 9,970 Rupees (SD = 12,600) compared to 2,550 Rupees (SD = 3,110) in Patna.

Interestingly, the monthly cost of TB medication was lower in Mumbai (114 Rupees, SD = 372) than in Patna (188 Rupees, SD = 660), suggesting better access to subsidized medications or differences in drug pricing between the cities. More details on perspectives on the COVID-19 pandemic, how participants utilized the health care during the COVID-19 pandemic as well as cost information are provided in the supporting information (Tables C-E in S1 Appendix).

### Individual care-seeking journeys during COVID-19

**Fig 3:**
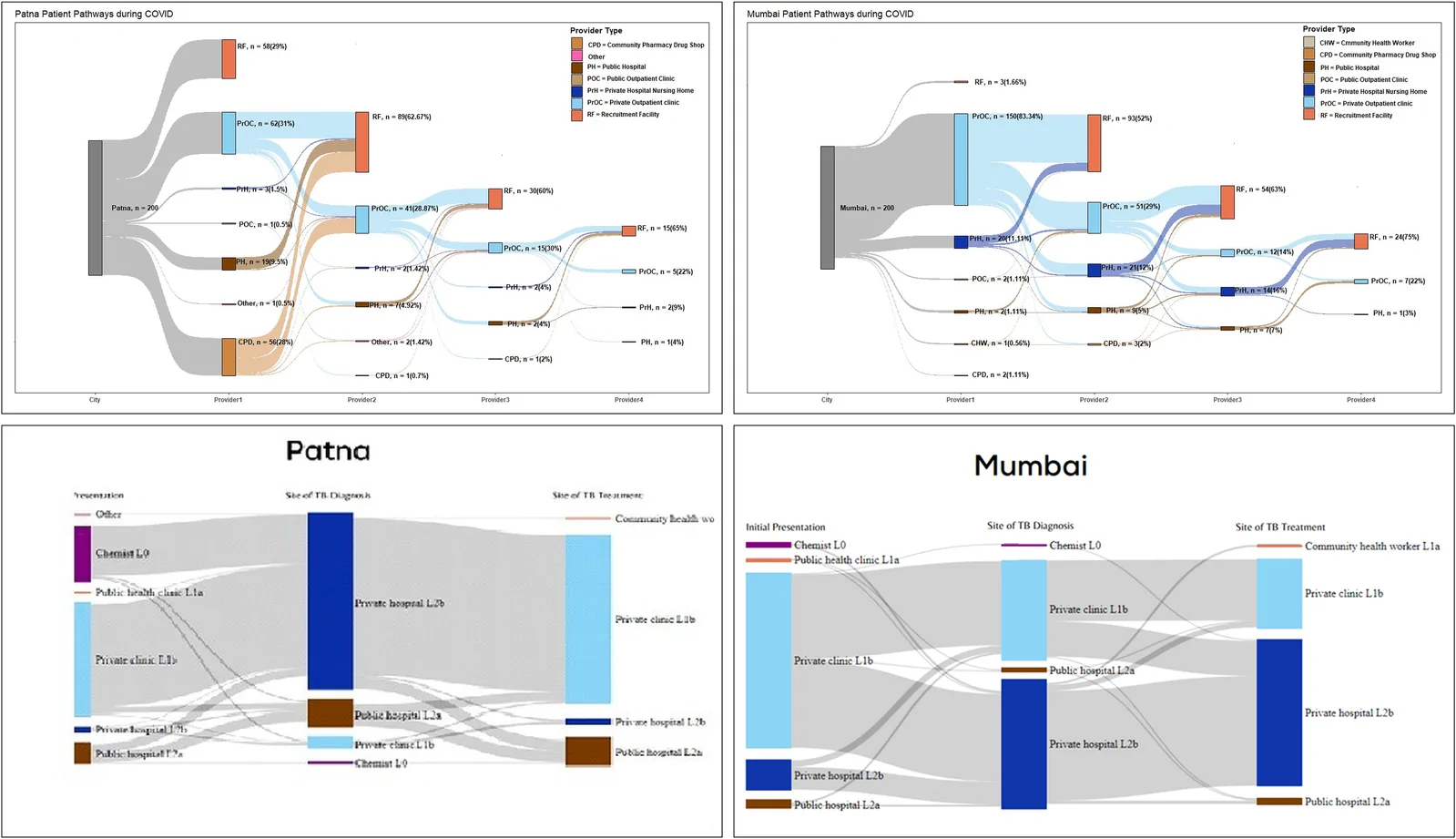
Sankey Diagrams (A - D) Showing Care-Seeking Journeys of People with TB in Patna and Mumbai During the COVID-19.

In Patna, initial care-seeking predominantly began at private outpatient clinics (31%), community pharmacies (28%), and recruitment facilities (29%). Participants often continued to visit private outpatient clinics or shifted between informal providers, such as pharmacies, reflecting a fragmented journey with multiple touch-points that could delay diagnosis and treatment. Public hospitals remained a minor player, accounting for only 9.5% of visits (Fig 3). In Mumbai, the care-seeking journeys were more streamlined, with 83% of participants initially choosing private outpatient clinics and 11% attending private hospitals or nursing homes. Subsequent visits often involved the same private providers.

In Patna, the initial healthcare encounter for TB symptoms predominantly occurred at private outpatient clinics (58%) and community pharmacies or chemists (28%), with only 11% seeking care in public hospitals. Similarly, in Mumbai, the majority of initial encounters were at private outpatient clinics (82%), followed by private hospitals (14%). The site of TB diagnosis showed a similar reliance on private facilities in both cities. In Patna, 86% of TB diagnoses were made at private outpatient clinics, whereas in Mumbai, private hospitals accounted for 55% of diagnoses, followed by private outpatient clinics at 44%.

For the initiation of TB treatment, Patna showed a strong preference for private outpatient clinics (85%), with fewer participants being treated at public hospitals (12%). In contrast, Mumbai had a higher reliance on private hospitals as the predominant site for treatment initiation (66%), indicating a greater reliance on these facilities compared to Patna. More details on patient journey and diagnostic test are provided in the supporting information (Table B in S1 Appendix).

### Missed opportunities for TB diagnosis

**Fig 4:**
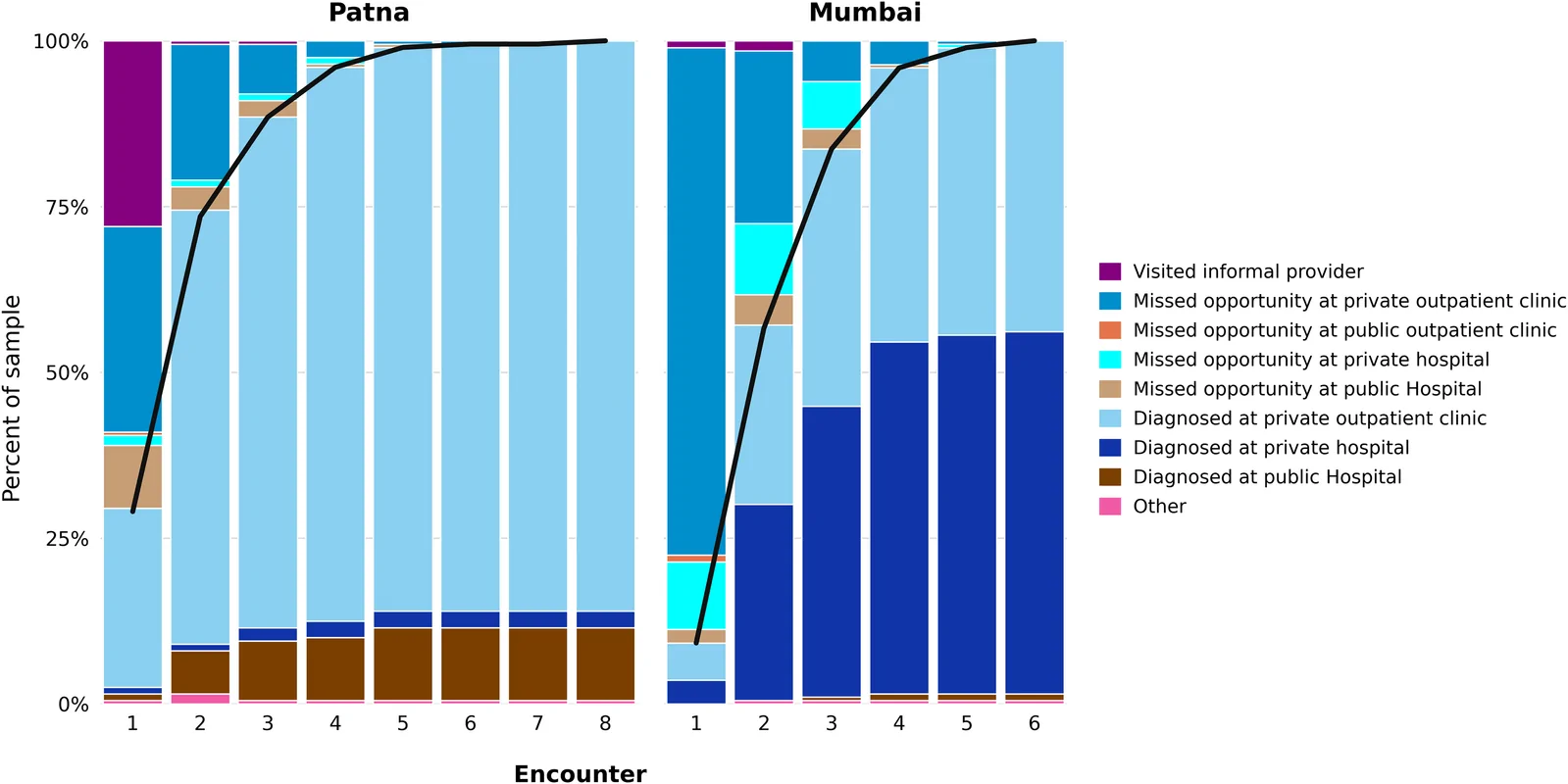
Bar charts showing sequential encounters in Patna and Mumbai during the COVID-19 pandemic.

Fig 4 illustrates the number of healthcare visits that were missed opportunities for a confirmed TB diagnosis in Patna and Mumbai. By the second or third encounter, 75% of people with TB in both cities had received a diagnosis of TB. However, private outpatient clinics were associated with a higher rate of missed diagnoses, contributing to 15% of missed diagnostic opportunities. In Patna, the frequency of missed diagnoses was slightly higher, with participants requiring an average of 3 visits before diagnosis. By the third visit, 28% of people with TB remained undiagnosed, a higher rate than in Mumbai. Public hospitals played a smaller role in both cities, representing only 2.5% of diagnostic opportunities in Mumbai and 9.5% in Patna.

### TB diagnostic testing across healthcare providers

**Fig 5:**
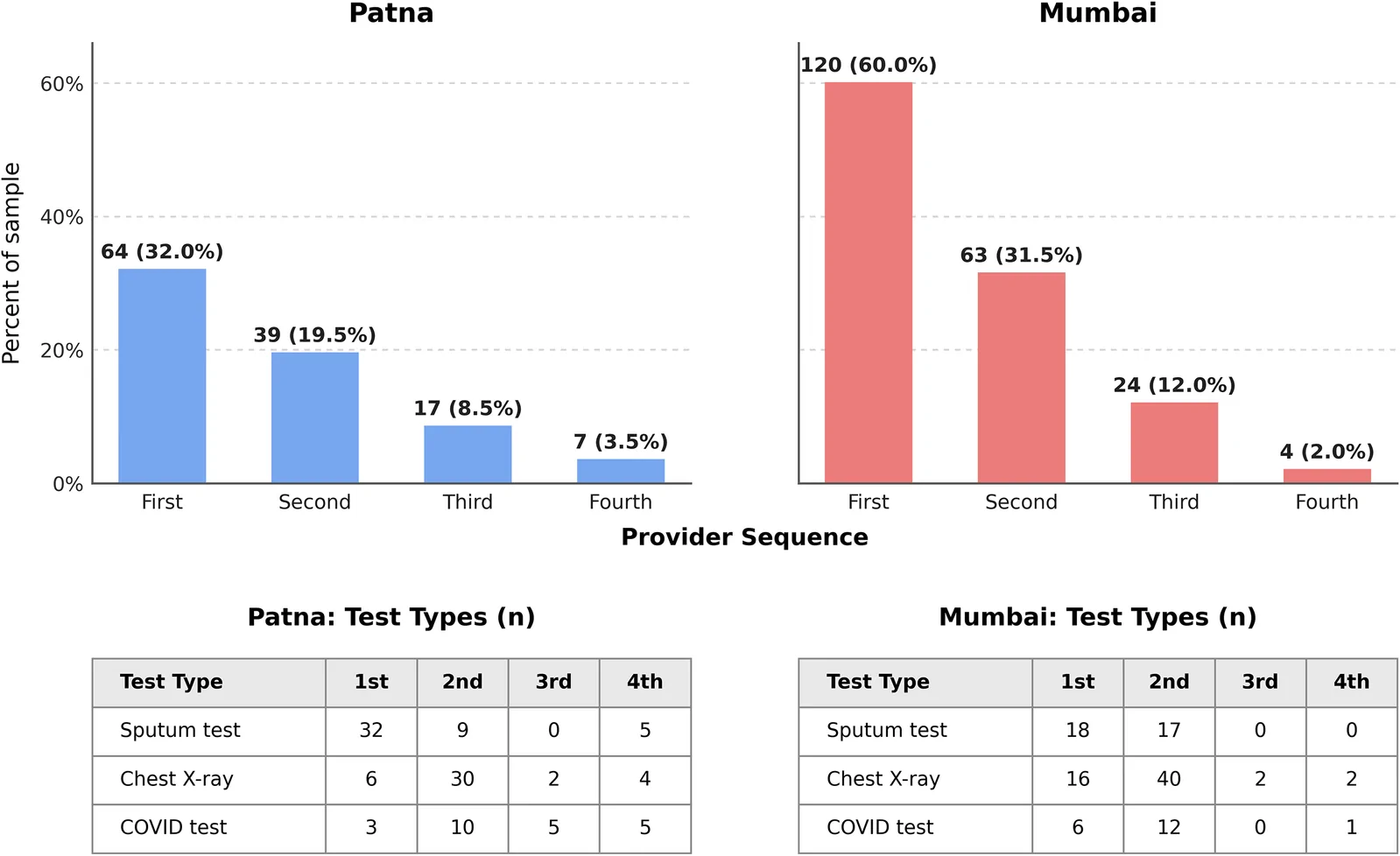
TB diagnostic patterns across sequential healthcare providers.

Fig 5 provides an overview of TB diagnostic testing patterns among various healthcare providers in Patna and Mumbai during the COVID-19 pandemic. In Patna, 32% of first providers suggested lab tests, whereas a higher proportion of providers in Mumbai (60%) recommended these tests at initial encounters. The types of tests varied significantly; sputum tests were initially recommended more frequently in Patna (50%) but were less common in Mumbai at first (15%), which also decreased in later visits, reaching 0% by the fourth provider. In contrast, chest X-rays were consistently recommended in Mumbai, with 13% of first providers and 63.35% of second providers suggesting this diagnostic tool.

Participants in Patna reported fewer difficulties in accessing recommended tests, with over 90% able to complete the tests without major barriers. In Mumbai, participants encountered more challenges over successive provider visits; while only 55.5% were able to complete tests without difficulty at their first encounter, this percentage declined further in subsequent visits. These challenges were primarily driven by prolonged waiting times (reported by 21.5% in Mumbai), the requirement for mandatory COVID-19 screening before TB diagnostic services could be accessed (noted by 66% of participants), and the high cost of laboratory tests, which averaged 9,970 Rupees in Mumbai—nearly four times the cost in Patna. Sputum samples were predominantly collected at public facilities in both cities, though 23.44% of samples in Patna were also collected in private clinics or labs during the initial encounters. The timeline for receiving sputum test results differed between the cities. In Patna, a greater proportion of participants (20.32%) received results within 1–2 days, compared to only 6% in Mumbai.

### Factors associated with diagnostic and treatment delays

**Fig 6:**
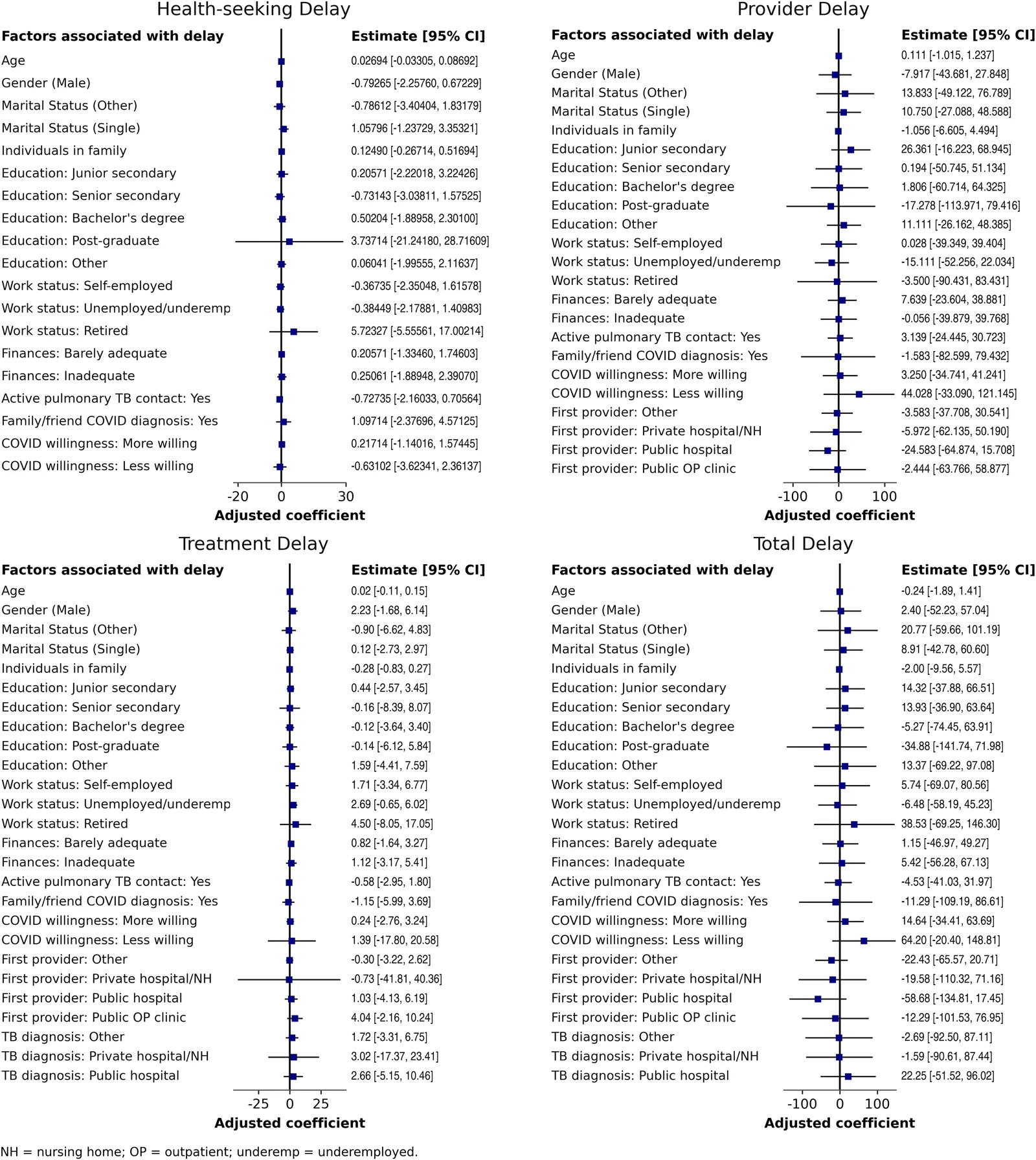
Factors associated with diagnostic and treatment delays during the COVID-19 pandemic in Patna.

**Fig 7:**
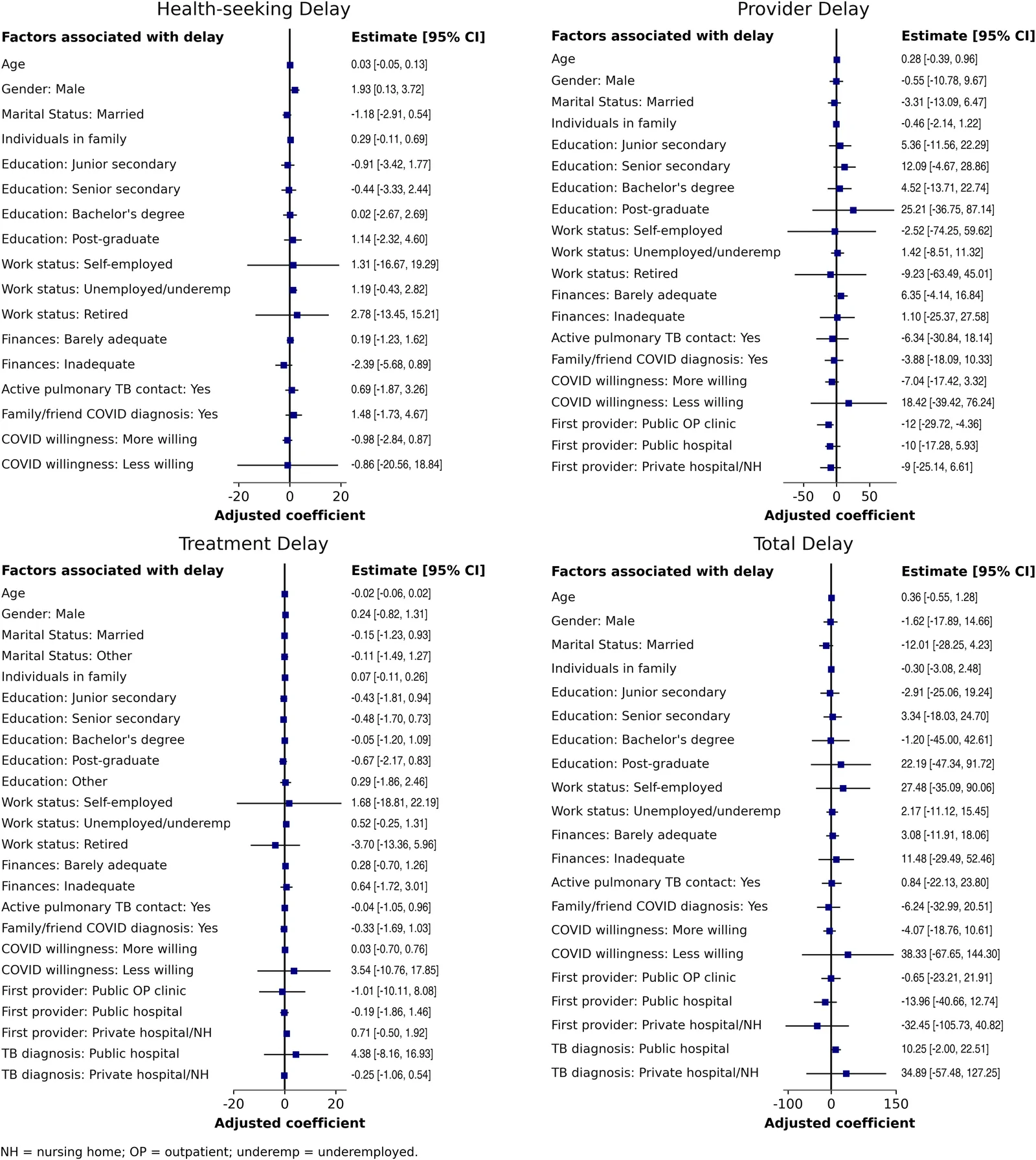
Factors associated with Diagnostic and treatment delays during the COVID-19 pandemic in Mumbai.

Fig 6 and Fig 7 presents the analysis of the factors associated with median change in healthcare-seeking, provider, treatment, and total delays (measured in days) during the COVID-19 pandemic in Patna and Mumbai. In Patna, for healthcare-seeking delay, males reported slightly shorter delay compared to females (adjusted median: -0.79 days; 95% CI: -2.26, 0.67; p = 0.290), and individuals with inadequate financial resources showed negligible difference (adjusted median: 0.25 days; 95% CI: -1.89, 2.39; p = 0.819). Provider delay was lower among males (adjusted median: -7.92 days; 95% CI: -43.68, 27.85; p = 0.665) and those who sought care at public hospitals (24.58 days, 95% CI: -64.87, 15.71; p = 0.234), though these differences were not statistically significant. Treatment delays were higher among males (adjusted median: 2.23 days; 95% CI: -1.68, 6.14; p = 0.266), while marital status and education level showed no notable influence. Total delay was slightly lower among males (adjusted median: 2.40 days; 95% CI: -52.23, 57.04; p = 0.931) and marginally shorter among individuals with bachelor’s degrees (adjusted median: -5.27 days; 95% CI: -74.45, 63.91; p = 0.882). Details of the unadjusted and adjusted analyses are presented in the supporting information (Tables F-M in S1 Appendix).

In Mumbai, men experienced longer delays in seeking care than women (adjusted median: 1.93 days; 95% CI: 0.13, 3.72; p = 0.037). Household size showed a trend toward increased delays, with a 0.29-day increase (95% CI: -0.11, 0.69; p = 0.080). Health seeking delays were also higher among unemployed individuals (adjusted median: 1.19; 95% CI: -0.43,2.82; p = 0.152) and those with adequate financial resources compared to those with inadequate means (adjusted median: -2.39 days; 95% CI: -5.68, 0.89; p = 0.155). In the case of provider delay, being married reduced this delay compared to single individuals (adjusted median: -3.31, 95% CI: -13.09,6.47; p = 0.508). Unemployed people had higher provider delays than those employed (adjusted median: 1.42; 95% CI: -8.51,11.32; p = 0.782), while initial visits to public outpatient clinics were associated with a decrease in provider delay (adjusted median: -12, 95% CI: -29.72, 4.36; p = 0.871).Treatment delay was marginally higher among males (adjusted median: 0.24, 95% CI: -0.82, 1.31; p = 0.232), and unemployed individuals (adjusted median: 0.52, 95% CI: -0.25, 1.31; p = 0.194), though these differences were not statistically significant. For total delay, gender, marital status, and the household size showed no significant impact; however, unemployed individuals faced a slightly higher delay (adjusted median: 2.17; 95% CI: -11.12,15.45; p = 0.777). A greater delay was observed among those less willing to seek care (adjusted median: 38.33; 95% CI: -67.65,144.30; p = 0.401).

### Factors associated with number of healthcare encounters

In Patna, gender and marital status were not significantly associated with healthcare encounters. Participants with junior school education or those studying for a bachelor’s degree reported fewer encounters compared to those with only primary education (adjusted coefficient: -0.47, 95% CI: -0.91, -0.02; p = 0.039) and those with secondary education (adjusted coefficient: -0.58, 95% CI: -1.19, 0.03; p = 0.060), respectively. Employment status was not a strong predictor, while those reporting inadequate resources showed a non-significant trend toward a greater number of encounters (adjusted coefficient: 0.34, 95% CI: -0.15, 0.83; p = 0.168).

The distance to the nearest public healthcare facility had no significant impact on the number of encounters. In contrast, a greater distance to private healthcare facilities was associated with a potential increase in encounters (adjusted coefficient: 0.64, 95% CI: -0.29, 1.56; p = 0.177). The frequency of internet use for health information and COVID-19-related factors, such as contact with people with active TB or having family or friends diagnosed with COVID-19, was not significantly associated with the number of healthcare encounters. However, those who were less willing to seek healthcare due to COVID-19 showed a trend toward an increased number of encounters (adjusted coefficient: 0.50, 95% CI: -0.10, 1.10; p = 0.100).

In Mumbai, a larger household size was associated with an increased number of healthcare interactions (adjusted coefficient: 0.08, 95% CI: 0.02, 0.14; p = 0.009). Unemployed participants had fewer encounters (adjusted coefficient: -0.38, 95% CI: -0.72, -0.05; p = 0.025), while individuals with barely adequate resources had an increased number of encounters (adjusted coefficient: 0.35, 95% CI: 0.02, 0.67; p = 0.038) (Table 4).

**Table 4:** Linear regression: factors associated with the number of encounters in Mumbai and Patna.

Variables	Patna (N = 200)		Mumbai (N = 200)	
Outcome: Number of encounters	Unadjusted Coefficient (95% CI), p-value	Adjusted Coefficient (95% CI), p-value	Unadjusted Coefficient (95% CI), p-value	Adjusted Coefficient (95% CI), p-value
**Gender**				
Female	Reference	Reference	Reference	Reference
Male	-0.02 (-0.34, 0.30), 0.898	-0.03 (-0.42, 0.35), 0.865	-0.06 (-0.34, 0.22), 0.679	-0.18 (-0.51, 0.15), 0.283
**Marital Status**				
Single	Reference	Reference	Reference	Reference
Married	-0.05 (-0.40, 0.30), 0.769	-0.09 (-0.48, 0.31), 0.668	-0.11 (-0.39, -0.16), 0.428	-0.20 (-0.51, -0.10), 0.193
Other	0.12 (-0.39, 0.64), 0.646	0.15 (-0.39, 0.70), 0.576	-0.16 (-0.99, 0.68), 0.713	-0.16 (-1.01, 0.70), 0.715
**Number of individuals in the house**	0.04 (-0.02, 0.10), 0.228	0.05 (-0.02, 0.11), 0.172	**0.08 (0.02, 0.13), 0.012**	**0.08 (0.02, 0.14), 0.009**
**Highest education level**				
Primary 6 or lower	Reference	Reference	Reference	Reference
Junior secondary school	**-0.57 (-0.99, -0.15), 0.008**	**-0.47 (-0.91, -0.02), 0.039**	0.20 (-0.24, -0.64), 0.371	-0.16 (-0.63, 0.31), 0.511
Senior secondary school	-0.06 (-0.65, 0.53), 0.838	0.13 (-0.53, 0.79), 0.700	-0.03 (-0.46, -0.41), 0.909	-0.21 (-0.71, 0.28), 0.391
Bachelor’s degree	-0.45 (-0.94, 0.04), 0.074	-0.58 (-1.19, 0.03), 0.060	0.04 (-0.36, 0.49), 0.871	-0.29 (-0.82, 0.24), 0.287
Post-graduate certificate	-0.34 (-1.12, 0.44), 0.393	-0.20 (-1.07, 0.67), 0.653	0.37 (-0.36, 1.10), 0.319	0.17 (-0.64, 0.97), 0.687
**Employment Status**				
Working	Reference	Reference	Reference	Reference
Self-employed	-0.28 (-0.78, 0.21), 0.261	-0.19 (-0.70, 0.31), 0.451	-0.33 (-1.10, 0.43), 0.393	-0.41 (-1.18, 0.36), 0.301
Unemployed/underemployed	-0.03 (-0.39, 0.33), 0.852	-0.07 (-0.50, 0.36), 0.758	-0.23 (-0.51, 0.05), 0.106	**-0.38 (-0.72, -0.05), 0.025**
**Financial Standing**				
Adequate	Reference	Reference	Reference	Reference
Barely adequate	0.11 (-0.24, 0.46), 0.532	0.14 (-0.23, 0.51), 0.464	0.21 (-0.07, 0.50), 0.140	**0.35 (0.02, 0.67), 0.038**
Inadequate	0.39 (-0.05, 0.82), 0.083	0.34 (-0.15, 0.83), 0.168	0.11 (-0.41, 0.62), 0.679	0.31 (-0.27, 0.90), 0.292
**Nearest Public Healthcare**				
Less than 2 km away	Reference	Reference	Reference	Reference
Between 2–10 km away	0.01 (-0.31, 0.34), 0.928	-0.11 (-0.47, 0.24), 0.538	-0.08 (-0.44, 0.28), 0.665	-0.28 (-0.68, 0.12), 0.166
**Nearest Private Healthcare**				
Less than 2 km away	Reference	Reference	Reference	Reference
Between 2–10 km away	**0.88 (0.04, 1.72), 0.041**	0.64 (-0.29, 1.56), 0.177	-0.31 (-1.13, 0.51), 0.457	-0.07 (-0.98, 0.84), 0.882
**Internet use for healthcare**				
Daily	Reference	Reference	Reference	Reference
Once a week	-0.13 (-1.38, 1.13), 0.844	0.08 (-1.26, 1.42), 0.908	**1.50 (0.04, 2.96), 0.044**	1.23 (-0.27, 2.73), 0.107
Less than once a week but more than once a month	0.71 (-0.57, 2.00), 0.275	0.92 (-0.46, 2.30), 0.191	-0.45 (-1.36, 0.45), 0.323	-0.69 (-1.68, 0.30), 0.172
Once a month or less	0.62 (-0.50, 1.75), 0.275	0.78 (-0.42, 1.98), 0.200	-0.20 (-1.00, 0.60), 0.621	-0.60 (-1.49, 0.29), 0.182
Never	0.10 (-0.90, 1.10), 0.847	0.13 (-0.92, 1.19), 0.800	-0.35 (-1.10, 0.39), 0.353	-0.82 (-1.65, 0.02), 0.055
**Friends or Family COVID-diagnosed**				
No	Reference	Reference	Reference	Reference
Yes	0.01 (-0.46, 0.48), 0.962	0.02 (-0.54, 0.59), 0.931	-0.03 (-0.39, 0.32), 0.848	-0.07 (-0.49, 0.34), 0.721
**COVID affected willingness to seek healthcare**				
No effect	Reference	Reference	Reference	Reference
More willing	-0.12 (-0.46, 0.22), 0.491	-0.07 (-0.43, 0.28), 0.688	0.02 (-0.25, 0.30), 0.874	0.03 (-0.28, 0.34), 0.859
Less willing	**0.72 (0.19, 1.26), 0.009**	0.50 (-0.10, 1.10), 0.100	**0.86 (0.20, 1.52), 0.011**	**0.89 (0.15, 1.64), 0.019**

A greater distance to both public and private healthcare was associated with lower numbers of encounters (adjusted coefficient: -0.28, 95% CI: -0.68, 0.12; p = 0.166) and (adjusted coefficient: -0.07, 95% CI: -0.98, 0.84; p = 0.882), respectively. Weekly internet use for health information was associated with a non-significant increase in encounters (adjusted coefficient: 1.23, 95% CI: -0.27, 2.73; p = 0.107). While contact with people with active pulmonary TB, having friends or family diagnosed with COVID-19, and varying willingness to seek healthcare due to COVID-19 were not significant, those reporting a lower willingness to seek care showed a slight, significant increase (adjusted coefficient: 0.89, 95% CI: 0.15, 1.64; p = 0.019) (Table 4).

## Discussion

The convergence of the TB epidemic and the COVID-19 pandemic posed dual challenges for healthcare systems, especially in resource-limited settings like India, creating unprecedented strain. Our study examined how health-seeking journeys of people with TB, healthcare access and associated costs in Mumbai and Patna, were affected by the COVID-19 pandemic. The results offer critical insights into care delays, patient behaviors, healthcare utilization patterns, and financial challenges, underscoring the urgent need for targeted public health measures to mitigate these adverse effects and build more resilient health systems for the future.

### Delays in care and factors influencing healthcare seeking behavior

Healthcare seeking behaviors during crises are significantly influenced by demographic and socioeconomic factors, as documented across multiple settings [49]. Gender-based disparities in care access during the pandemic have been observed globally, with several studies noting shifts in typical patterns [50]. In Mumbai, men experienced longer delays compared to women, reflecting gender disparities consistent with cultural norms and previous research [49]. Larger household sizes in Mumbai were associated with increased healthcare interactions, possibly due to heightened vigilance among families at risk. Research from other high-burden settings similarly suggests that the pandemic altered usual care-seeking behaviors, potentially exacerbating pre-existing inequities in healthcare access. The notably short health-seeking delay observed in our study (0 days in Patna; 3 days in Mumbai), markedly shorter than the 1–2-month delays reported in pre-pandemic Indian literature, warrants specific interpretation. We argue this reflects a pandemic-specific phenomenon rather than a pre-existing high level of TB awareness. The overlapping symptom profiles of COVID-19 and TB (cough, fever, fatigue), combined with intense public health messaging urging immediate care-seeking for respiratory symptoms, likely ‘pulled forward’ help-seeking among people with TB who might otherwise have delayed. This interpretation is supported by our finding that 50.5% of Mumbai and 31.5% of Patna participants reported being more willing to seek care specifically because of the pandemic. The low-barrier accessibility of community pharmacies as a first contact point further facilitated near-immediate care-seeking independent of any specific suspicion of TB [51].

Unemployment contributed to longer delays in both cities, suggesting that financial insecurity during the pandemic hampered timely access to care. The complex relationship between economic resources and healthcare utilization during crises has been extensively documented by multiple researchers, who have consistently found that individuals with extremely limited resources often forgo care entirely [52]. In contrast, those with constrained but not absent resources may make extraordinary efforts to access healthcare despite significant barriers. Interestingly, individuals with limited but adequate resources sought more healthcare encounters, possibly driven by a heightened perceived risk despite economic constraints. This paradoxical trend underscores the importance of financial security in healthcare utilization [47,53]. Most participants with unstable employment still sought care in the private sector, not by choice, but because it was the most accessible option. Private providers were nearby for 94–96% of participants, while only 63.5% in Patna lived within the same distance of a public facility. On top of this, 14.5% of Patna participants reported that public facilities had been closed or redirected to COVID-19 care during the pandemic. Community pharmacies, the first point of contact for 41% of Patna participants, offered a low-barrier starting point, with more costly hospital visits following only when symptoms worsened.

When we looked at what predicted longer delays, no single individual characteristic (sex, age, employment, or education) stood out as a consistent driver. It suggests that delays are not really about who the patient is; they reflect how the system is structured. Providers operate in silos, referral pathways between them are inadequate, and while diagnostic services are available for free in the public sector, patients who enter through private providers are rarely linked across to where that free care exists. The implication is that reducing delays requires fixing the system through standardised diagnostic pathways, stronger public–private linkage, and clearer referral accountability, and not targeting specific groups with messaging.

### Impact of healthcare provider type on diagnostic and treatment delays

The role of private healthcare providers in care delays during emergencies has been increasingly recognized in health systems research [54]. Studies from several South Asian contexts have documented how reliance on private providers shapes care journeys, particularly in urban settings [18]. This phenomenon is especially pronounced in India, where reliance on private healthcare reflects longstanding perceptions about quality and accessibility but creates substantial financial vulnerability during crises [38].

The predominance of private healthcare providers played a significant role in care delays. In Patna, initial visits to public hospitals were associated with shorter provider delays. In contrast, Mumbai’s reliance on private hospitals resulted in higher consultation fees and potential delays due to overwhelmed capacity. This reliance on private healthcare, especially in Mumbai, reflects perceptions of better quality and proximity but has led to substantial patient financial burdens [5], this reliance on private healthcare during the pandemic resulted in substantial financial burdens, with people with TB facing high out-of-pocket expenses [54,55]. The disparities in diagnostic costs between the cities further compounded challenges, particularly for lower-income households disproportionately affected by increased healthcare expenses [8].

### Economic impact of COVID-19 on people with TB

The pandemic’s economic impact on healthcare costs has been widely documented. Research from multiple settings has demonstrated how health emergencies can exacerbate the economic burden of chronic disease care [55], with particularly severe effects on socioeconomically vulnerable populations [8]. Studies from Nigeria and Peru have similarly documented how financial barriers during the COVID-19 pandemic delayed healthcare access and treatment adherence for people with TB [46], suggesting this is a widespread phenomenon that requires policy attention.

The economic burden of TB care intensified during the COVID-19 pandemic, particularly in Mumbai, where 85.5% of participants reported higher healthcare costs driven by elevated consultation and diagnostic fees. These financial strains likely delayed healthcare access and treatment adherence, echoing trends seen in other settings during the pandemic [46]. Policies to reduce out-of-pocket expenses, such as expanding public healthcare subsidies or providing financial aid for diagnostics, could mitigate these barriers.

### Policy implications

The COVID-19 pandemic offers important lessons about health system vulnerabilities worldwide. Global health experts have noted how the pandemic exposed critical weaknesses in essential service delivery that extend beyond the acute crisis period [56]. The documented disruptions to TB care during COVID-19 mirror patterns seen during previous health emergencies, suggesting the need for fundamental strengthening of health service delivery models [57].

Building more resilient health systems requires addressing several key dimensions. Research on health system strengthening emphasizes the importance of integrating public and private healthcare sectors to ensure coordinated responses during crises [38] and implementing financial protection mechanisms that prevent catastrophic expenditures [58]. The WHO and the Stop TB Partnership have highlighted the importance of developing emergency preparedness plans specifically for maintaining essential services, which can protect vulnerable populations during future crises [59]. Evidence from multiple settings suggests that addressing socioeconomic and gender-based barriers through targeted interventions is crucial for building equitable and resilient systems capable of withstanding future shocks [57].

Our findings underscore the need for policy interventions to strengthen TB services and prepare health systems for future disruptions. Strengthening public healthcare systems, particularly in high-burden urban areas like Mumbai, can reduce reliance on costly private care and improve access to affordable health services. Integration between the public and private sectors and investments in public healthcare infrastructure can foster a more resilient system capable of managing concurrent health emergencies [60]. Reducing out-of-pocket expenses, such as subsidizing medications and diagnostic tests and enhancing public health insurance, would financially relieve vulnerable populations. Addressing gender-specific barriers, as evidenced by longer delays among men in Mumbai, is another priority. Public health campaigns tailored to cultural norms can promote equitable healthcare access and improve diagnostic timeliness.

Investment in digital health solutions, including telemedicine and mobile health (mHealth) tools, has been increasingly recognized as a strategy to bridge gaps in healthcare access [61,62]. By leveraging these technologies for patient education and remote monitoring, health systems can maintain the continuity of TB care even when in-person services are disrupted [61,62]. By promoting digital literacy and expanding telehealth services, policymakers can enhance healthcare accessibility, reduce delays, and minimize costs during emergencies. Leveraging these tools for patient education and monitoring will ensure continuity of care even in times of crisis.

### Limitations

This study’s strengths include its comprehensive analysis of patient journeys, the economic impact of healthcare costs, and factors influencing healthcare interactions during the COVID-19 pandemic. Using both quantitative and qualitative data enabled a nuanced understanding of the challenges faced by people with TB in Mumbai and Patna. However, there are limitations to consider. The cross-sectional design limits the ability to establish causality, and the self-reported nature of some variables may introduce recall bias. In particular, Mumbai participants were interviewed later (December 2022–January 2023) than those in Patna (February–March 2022), which may have introduced differential recall bias in the reconstruction of care-seeking timelines. While we standardized the eligibility period to 2020–2022 and field officers used structured calendar tools and national lockdown dates as memory anchors, residual bias from differential recall periods cannot be fully excluded. Additionally, the study focused on two urban centers, which may not to be generalizable to rural areas where healthcare access and economic conditions differ significantly. The potential for recall bias in self-reported healthcare-seeking behaviors has been acknowledged in similar studies [38,62] and cross-sectional designs have inherent limitations for establishing causal relationships. Additionally, the generalizability of urban-focused research to rural areas requires careful consideration, though the patterns observed often highlight important systemic issues relevant to broader TB control efforts.

## Conclusion

Our study underscores the complexities and challenges people with TB faced in India during the COVID-19 pandemic, highlighting the critical need for adaptive, patient-centered healthcare systems. The COVID-19 pandemic revealed vulnerabilities in TB care journeys in urban India that provide important lessons for health system resilience beyond the immediate crisis. Strengthening the integration between public and private healthcare sectors, reducing financial barriers, leveraging technology for healthcare delivery, and promoting equitable access to care is imperative. By positioning COVID-19’s impact as a case study of health system disruption, we demonstrate how diagnostic, and treatment delays reflect structural weaknesses that persist beyond the acute crisis period. This study contributes valuable insights into the complexities of managing TB during health system disruptions, offering directions for future research and policy-making that mitigate the impact of concurrent health crises on vulnerable populations and strengthen system-wide preparedness for maintaining essential services during future emergencies. Three findings stand out for policy and practice. First, delays were predominantly system-driven: once people sought care, structural fragmentation, and not patient hesitancy, was the main barrier. Second, the fragmented private sector generated multiple unnecessary pre-diagnosis encounters and substantial out-of-pocket costs, patterns that predate COVID-19 and will persist without deliberate reform. Third, no individual characteristic consistently predicted delay, which points to the system rather than the patient as the problem. Together, these findings make the case for public–private integration, financial protection, and standardised diagnostic pathways as routine components of urban TB care, not emergency measures.

## Supporting information

S1 Appendix Supporting information for ‘Delays in tuberculosis diagnosis and treatment in India: A patient journey analysis of people with TB from Mumbai and Patna during the COVID-19 pandemic’. (DOCX)
